# Comparison of Three Extraction Approaches for the Isolation of Neurotransmitters from Rat Brain Samples

**DOI:** 10.3390/ijms19061560

**Published:** 2018-05-24

**Authors:** Natalia Miękus, Ilona Olędzka, Darya Harshkova, Ivan Liakh, Alina Plenis, Piotr Kowalski, Tomasz Bączek

**Affiliations:** 1 Department of Animal and Human Physiology, Faculty of Biology, University of Gdańsk, Wita Stwosza 59, 80-308 Gdańsk, Poland; 2 Department of Pharmaceutical Chemistry, Medical University of Gdańsk, al. Gen. J. Hallera 107, 80-416 Gdańsk, Poland; ilona@gumed.edu.pl (I.O.); aplenis@amg.gda.pl (A.P.); piotr.kowalski@gumed.edu.pl (P.K.); tbaczek@gumed.edu.pl (T.B.); 3 Department of Plant Physiology and Biotechnology, University of Gdańsk, Wita Stwosza 59, 80-308 Gdańsk, Poland; darya.gorshkova@gmail.com; 4 S. I. Gelberg Department of Microbiology, Virology and Immunology, Grodno State Medical University, Vilenskaja str., 19, 230023 Grodno, Belarus; liakh_ivan@mail.ru

**Keywords:** brain cortex slices, neurotransmitters, micellar electrokinetic capillary chromatography

## Abstract

The determination of neurotransmitters (NTs) as relevant potential biomarkers in the study of various central nervous system (CNS) pathologies has been demonstrated. Knowing that NTs-related diseases mostly occupy individual regions of the nervous system, as observed, for instance, in neurodegenerative diseases (Alzheimer’s and Parkinson’s Diseases), the analysis of brain slices is preferred to whole-brain analysis. In this report, we present sample preparation approaches, such as solid-phase extraction, solid-phase microextraction, and dispersive liquid–liquid microextraction, and discuss the pitfalls and advantages of each extraction method. The ionic liquid (1-ethyl-3-methylimidazolium tetrafluoroborate)-assisted solid-phase microextraction (IL-SPME) is found to be, in our research, the relevant step towards the simultaneous determination of six NTs, namely, dopamine (DA), adrenaline (A), noradrenaline (NA), serotonin (5-HT), l-tryptophan (l-Trp), l-tyrosine (l-Tyr) in rat brain samples. The development of a novel bioanalytical technique for the evaluation of biomarkers in the context of green chemistry might be accelerated just with the use of IL, and this approach can be considered an advantageous strategy.

## 1. Introduction

Preclinical discoveries are often not reproducible, in part because of the inadequate cell lines and animal models applied for such studies [1]. Most of the relevant achievements in medicine (also among Nobel Price laureates) were and still are acquired on animal models of human diseases [2,3]. The different brain initiatives are believed to gather interdisciplinary scientists who work as a team and complement each other, which is critical for facilitating progress in the understanding of brain function. There are already outcomes of projects since the development of advanced cerebral organoids that mimic the human brain. These organoids are used as a new platform to investigate human brain development [3]. While waiting for further improvements of these organoids to study complex central nervous system (CNS) diseases, brain sample analytical platforms require improvement and development [4]. The use of an optimized sample preparation method prior to micellar electrokinetic chromatography with spectrophotometric detection (MEKC–UV/VIS) as an alternative to the expensive enzyme immunoassays for the research of disturbances in cell signaling, opens up new possibilities for the study of complex relationships and interactions between neurotransmitters (NTs) and their precursors and metabolites in pathologies of the CNS. The proposed biomarker profile can be helpful in the early diagnosis and monitoring of neurodegenerative diseases in humans, such as Alzheimer’s and Parkinson’s Diseases (AD, PD, respectively). An in-depth study of the basics of AD and PD could improve our knowledge of such pathologies [5,6,7].

The purpose of the presented statement was to demonstrate a straightforward and not too time-consuming sample preparation methodology for the analysis of NTs together with their precursor amino acids and metabolites in rat brain samples. We developed a methodology for the isolation of selected NTs from brain tissue using an ionic liquid (IL) in order to increase the efficiency of solid-phase microextraction (SPME) for the selective extraction of six NTs, namely, dopamine (DA), serotonin (5-HT), adrenaline (A), noradrenaline (NA), l-tryptophan (l-Trp), and l-tyrosine (l-Tyr). It should be noted that, after the discovery of ILs more than 100 years ago, their undoubted importance has been highlighted in various fields [8,9,10]. Since IL-based extraction techniques have the capability of isolating and enriching various organic and inorganic compounds, we implemented them in our brain sample preparation method development. We also identified the most appropriate medium for the homogenization of rat brain slices and the preconcentration of NTs prior to MEKC coupled with simple spectrophotometric detection of the analytes in the biological samples.

## 2. Results and Discussion

The amount of brain tissue was reduced to fulfill the requirements for the miniaturization of sample and solvents. The idea of the presented study was to discuss the most popular sample preparation approaches for the isolation of NTs, their precursor amino acids, and their metabolites from brain slices. To clearly demonstrate the pros and cons of the chosen analytical approaches, 200 mg of brain tissue sample was processed by mechanical homogenization with the most popular solvents (formic acid (FA) or perchloric acid (PCA) solutions) [11,12,13,14,15]. For the stability of the analytes, the homogenization was performed in a hand-made ice bath, and the entire experimental procedure was carried out in the dark. To evaluate a sample preparation method useful in everyday clinical practice and basic studies, the most accessible mechanical homogenizer and equipment for extraction were used.

**Figure 1:**
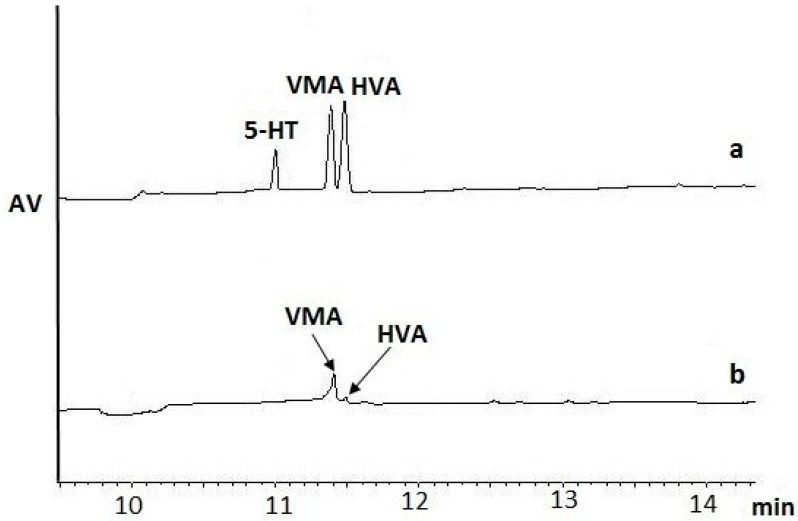
Comparison of two media for the homogenization step: perchloric (PCA) and formic acid (FA) solutions in methanol (PCA/MeOH vs. FA/MeOH). Electropherogram of brain samples spiked with nine neurotransmitters (NTs) (each analyte at the concentration of 10 µg/mL) obtained after homogenization with either FA/MeOH (**a**) or PCA/MeOH (**b**) solutions without further purification. The separation was carried out using the optimized micellar electrokinetic chromatography with spectrophotometric detection (MEKC–UV/VIS) method. Capillary electrophoresis (CE) conditions: uncoated fused silica capillary (75 µm i.d. (internal diameter) 60.2 cm length); λ = 200 nm; injection time 8 s; applied voltage 25 kV; temperature 25 (±0.1) °C. The background electrolyte (BGE) consisted of 10 mM sodium tetraborate decahydrate, 30 mM SDS, 15% (*v/v*) MeOH, and 25 mM alpha cyclodextrin (α-CD), adjusted to pH 9.36 using 1 M sodium hydroxide (NaOH). Legend: 5-HT—serotonin; VMA—vanillylmandelic acid; HVA—homovanillic acid.

We evaluated the performance of several experiments for the isolation, preconcentration, and determination of NTs in rat brains enriched with l-Tyr, levodopa (L-DOPA), DA, A, NA, homovanillic acid (HVA), vanillylmandelic acid (VMA), l-Trp, and 5-HT (study samples). The control samples consisted of brain slices with the addition of methanol (MeOH) instead of the analytes’ standard solution. Brain samples of 200 mg (control and study samples) were weighed and homogenized in a mixture 1:1 (*v/v*) volume of ice-cold 0.1% FA in MeOH or 3% PCA in MeOH. The obtained homogenates were centrifuged, and then the supernatant was immediately separated from the precipitate. The data showed that FA, as a homogenization medium, worked more efficiently for the HVA and VMA isolation step (Figure 1a) than PCA (Figure 1b) (Table 1). The FA/MeOH solution was superior to the FA/H_2_O solution for the isolation of NTs, as proved in previous studies performed by He et al. [12]. The isolation of 5-HT was also successful (Figure 1a, Table 1). The results of the extraction efficiency for the other studied NTs, i.e., DA, A, NA, l-Trp, l-Tyr, indicated that the amounts of NTs extracted were below the limits of detection (LOD) of the applied MEKC-UV/VIS-based method.

**Figure 2:**
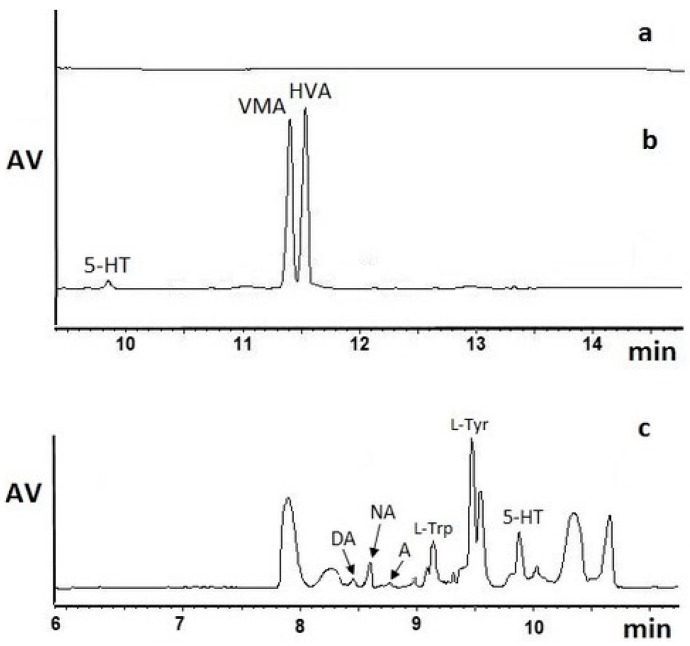
Electropherogram of (**a**) the blank brain control sample after the SPE SCX sample clean-up with 0.1% FA in ACN as the eluent, (**b**) the brain sample spiked with nine NTs (each analyte at the concentration of 10 µg/g) obtained after the SPE SCX sample clean-up with 0.1% FA in ACN/MeOH (1:1, *v/v*), (**c**) the brain sample spiked with nine NTs (each analyte at the concentration of 10 µg/mL) obtained after the SPE SCX sample clean-up with MeOH as the eluent. CE conditions as in Figure 1. Legend: DA—dopamine; A—adrenaline; NA—noradrenaline; l-Trp—l-tryptophan; l-Tyr—l-tyrosine; 5-HT—serotonin; VMA—vanillylmandelic acid; HVA—homovanillic acid.

To achieve better extraction yields, we planned some additional steps for the isolation, pre-concentration, and sample clean-up procedures of brain NTs. Three extraction methods were used: solid-phase extraction (SPE) using STRATA Strong Cation Exchange (SCX) or Clean Screen DAU (CSDAU) columns, dispersive liquid–liquid microextraction (DLLME), and solid-phase microextraction (SPME). The SPE coatings and eluents were chosen on the basis of previously published data related to the preparation of biological samples for the isolation of some NTs or their metabolites [15,16]. Comparing the already published data with our studies, the most appropriate SPE column conditioning, equilibration, and sample eluent media were chosen (Table 2). Indeed, our experiments proved that for acidic metabolites (VMA and HVA), isolation with the use of the SPE SCX-based method and 0.1% FA in acetonitrile (ACN)/MeOH as the elution solvent worked efficiently (Figure 2b, Table 1). SPE allowed to obtain purified brain samples that were enriched with VMA and HVA, since the signal intensities (the height of the analyte peaks) were 15 times higher than those obtained after the analysis of brain homogenates without the SPE procedure. Moreover, the baseline was free of interfering signals from the sample matrix (Figure 2a). On the other hand, a lower extraction efficiency was observed for 5-HT due to the basic physicochemical properties of this biogenic amine. The signal intensities of other basic compounds, such as A, NA, DA, l-Trp, l-Tyr, were below the LOD (Figure 2b).

To verify the influence of the changes in the SPE tunable parameters and the increase in the extraction efficiency of VMA and HVA, but also basic NTs, by SPE SCX, we changed the SPE eluent and applied pure MeOH or MeOH with sodium hydroxide (pH 9.3) for the elution of the analytes. In both cases, the efficiency was below our expectations, and the baseline was not stable because of additional signals coming from the matrix. Those signals interfered with the peaks of the studied compounds, which hindered the determination of basic NTs, as shown in Figure 2c (MeOH) and in Table 2. Indeed, pure MeOH as the SPE eluent of basic NTs demonstrated greater efficiency than MeOH with ammonium hydroxide (NH_4_OH).

Furthermore, the change in the SPE column type was studied towards the enhancement of the isolation of NTs from rat brain samples. The SPE SCX efficiency was based on the cation exchange mechanism, whereas the CSDAU columns utilized both a reverse (C8) phase and an ion exchange (benzenesulfonic acid) phase bonded to the same particle. The data obtained in our studies showed lower efficiency of CSDAU columns for the selective isolation of HVA and VMA. Additionally, the basic NTs, like A, NA, DA and amino acids (l-Tyr, l-Tryp), were not isolated efficiently enough using CSDAU columns to be detected by a diode array detector (DAD) coupled with the elaborated MEKC method. SPE CSDAU allowed 5-HT to be isolated, but the peak height was three times lower than that obtained when the clean-up step was omitted (Table 1).

**Figure 3:**
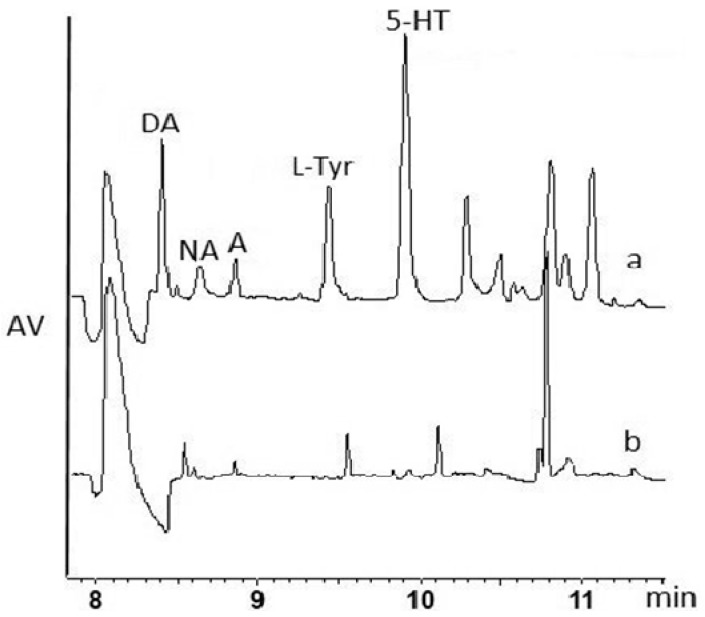
Electropherogram of DLLME for the isolation of NTs from (**a**) the brain sample spiked with nine NTs (each analyte at the concentration of 10 µg/g), (**b**) the blank brain control sample. Legend and CE conditions as in Figure 2.

In the past five years, some authors applied DLLME for brain sample preparation for the determination of NTs but they used rat brain microdialysates, and the derivatization of the analytes was also necessary [17,18]. We applied the DLLME-based method for the isolation of NTs from brain samples which was previously tested and optimized for the isolation of NTs from urine samples [19]. However, for the brain samples, we carried out the procedure for the first time. The conditions that were optimized for urine samples worked relatively well for the isolation of DA, A, NA, l-Tyr, 5-HT, whereas, for l-Trp, HVA, and VMA, this protocol was not appropriate (Figure 3a, Table 1). The application of DLLME provided a simple and fast enrichment of the sample containing DA, A, NA, l-Tyr, 5-HT, since the MEKC–DAD method allowed those analytes to be determined without the need for derivatization or modification of the detector (there was no need for more sensitive laser-induced fluorescence (LIF) or mass spectrometry (MS) detectors). On the other hand, the sample matrix could have an influence on the determination of the analytes (Figure 3) because brain samples are much more complex than urine.

**Figure 4:**
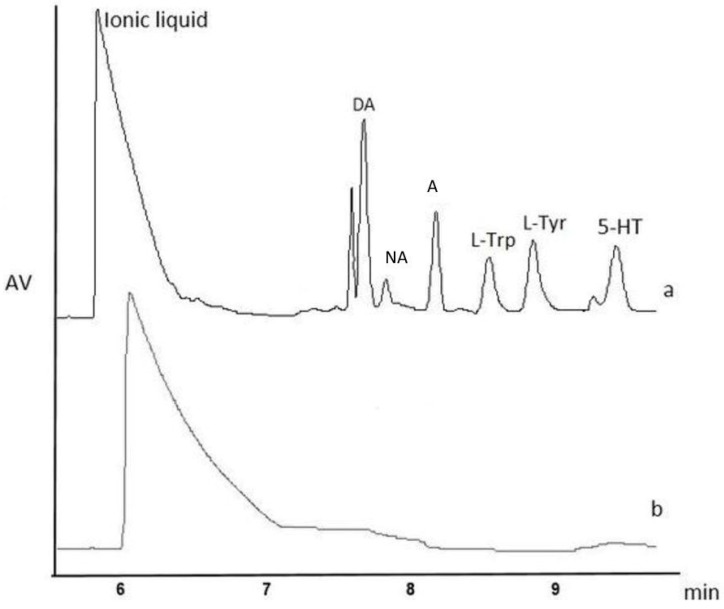
Electropherogram of SPME for the isolation of NTs from (**a**) the brain sample spiked with nine NTs (each analyte at the concentration of 10 µg/g); (**b**) the blank brain control sample. Legend and CE conditions as in Figure 2.

The last procedures that we have evaluated were based on the SPME technique with a polystyrene–divinylbenzene (PS–DVB) resin. Cudjoe et al. demonstrated the usefulness of the SPME procedure for the analysis of DA and 5-HT from 2.0 g of a rat brain sample [20]. Indeed, SPME is a powerful microextraction tool which cleans-up and concentrates up to 96 samples during one procedure. Thus, the time for the preparation of an individual sample decreases, and the entire experimental procedure consumes little time, which is relevant for easily degraded compounds such as NTs, their metabolites, and their precursor amino acids. Moreover, for this purpose, we applied, for the first time, IL, i.e., 1-ethyl-3-methylimidazolium tetrafluoroborate, to enhance the extraction efficiency of NTs from a 200 mg sample of rat brain. This IL was tested as an additive to the SPME desorption phase, as it was previously done for urine samples [9]. Figure 4 shows the electropherograms obtained after the analysis of rat brain homogenates purified and enriched with the newly invented IL–SPME assisted method. A shift in the migration times (MTs) of the studied compounds was observed, but the extraction yields of SPME in comparison to DLLME were improved two times for DA and 3.5 times for NA (Table 1). In the case of A and l-Tyr, the signal intensities were slightly higher, while, for 5-HT, SPME worked less efficiently than DLLME. Moreover, with IL–SPME, the determination of l-Trp was possible (Figure 4a, Table 1). The improvement in matrix component elimination was superior when SPME was used instead of DLLME (Figure 4b vs. Figure 3b). Although the signal from the IL added to the desorbent was relatively extensive, it did not interfere with the signals of the analytes.

When comparing the isolation of NTs using SPE SCX with MeOH, MeOH with NH_4_OH (pH 9.3), or SPE CSDAU, the IL–SPME-assisted procedure was found to be more effective, showing higher signal intensities of the analytes (Table 1). Moreover, it was possible to omit the derivatization step, which is often applied during the analysis of rat brain NTs [21], for three of our tested methods. Nevertheless, the isolation of HVA and VMA was only achieved when SPE SCX with 0.1% FA in ACN/MeOH as an eluent was carried out.

## 3. Materials and Methods

### 3.1. Equipment for the Extraction Procedures

The quantitative analysis of analytes was conducted with the capillary electrophoretic system (P/ACE MDQ Capillary Electrophoresis System, Beckman Instruments, Fullerton, CA, USA). For sample preparation, the homogenizer T10 ULTRA (IKA, Warsaw, Poland), scale: WAKS 60/160 (Radwag, Warsaw, Poland), centrifuge Megastar 600R (VWR, Chicago, IL, USA), vortex MS3 basic shaker (IKA, Warsaw, Poland), and centrivap concentrator ST-02202 (Labconco, Kansas City, MO, USA) were used. The Supel-Select SCX SPE (30 mg/1 mL) columns were purchased from Supelco (Darmstadt, Germany), whereas the Clean Screen CSDAU 303 columns (300 mg/3 mL) were obtained from United Chemical Technologies Inc. (Bristol, PA, USA). The 96-well solid-phase microextraction (SPME) procedure employed a polystyrene–divinylbenzene (PS-DVB) resin and a 2.2 mL polypropylene deepwell storage plate supplied by Pas Technologies (Magdala, Germany).

### 3.2. Reagents

All chemicals were of analytical grade and applied without further purification. DA, A, NA, l-Trp, l-Tyr, 5-HT, l-DOPA, HVA, VMA, and 1-ethyl-3-methylimidazolium tetrafluoroborate (IL) were purchased from Sigma (St. Louis, MO, USA). MeOH, NH_4_OH, ethanol, and acetone were delivered by POCh (Gliwice, Poland). Sodium dodecyl sulfate (SDS) was supplied by Bio-Rad (Hercules, CA, USA). Reagents: PCA, FA, dichloromethane (DCM), α-cyclodextrin (α-CD), and sodium tetraborate decahydrate (borax) were obtained from Merck (Darmstadt, Germany). Capillary Regenerator Basic Wash sodium hydroxide was purchased from Beckman Coulter (Brea, CA, USA). The water used in all experiments was obtained from Milli-Q equipment (Bedford, MA, USA).

### 3.3. Preparation of Stock and Working Solutions

Stock solutions of A, NA, L-DOPA, and l-Tyr (1.0 mg/mL) were prepared by dissolving the precise weighed portions of each standard (1.0 mg) in 950 µL MeOH and 50 µL 0.1 M HCl. Stock solutions of 5-HT, l-Trp, DA, HVA, and VMA (1.0 mg/mL) were prepared by accurately weighing 1.0 mg of each analyte in 1.0 mL of MeOH. The stock standard solutions were kept in a freezer (−20 °C), and fresh solutions were prepared once every two weeks. The working solutions were prepared daily, just before use, by diluting the stock solution as appropriate with deionized water. The working solutions were stored at 4 °C in a closed container for a maximum of 5 h.

### 3.4. Brain Sample Preparation

The analysis was carried out on brain samples obtained from healthy Wistar strain rats weighing approximately 250 ± 20 g and bought from a licensed breeding center (Tri-City Academic Laboratory Animal Centre of the Medical University of Gdansk). During the experiments, the animals lived in the Faculty of Biology Animal House, which meets the Polish Bills of experiments on animals from 21 January 2005 (Journal of Laws, 24 February 2005) as well as recommendations of the European Commission concerning the welfare of animals used in scientific experiments. The animals were housed 2–3 per cage, kept under a 12 h light–dark cycle (lights on at 6 a.m.) and permitted food and water ad libitum. The animal samples were collected by qualified scientists of the Department of Animal and Human Physiology following the rules of experimental procedures involving animals, pointed out by the Ethics Committee. The animals were deeply anesthetized with sodium pentobarbital (thiopental sodium, Biochemie GmbH, Hamburg, Germany; 80 mg/kg of body weight, i.p.) and sacrificed by decapitation with the use of a guillotine. Rapidly after the decapitation, the brains were taken out at low temperature (4 °C) to the clean probes, described, and kept in the freezer (−80 °C) until further analysis. The brain cortex slices were collected after decapitation by professional staff following the animal care and treatment guidelines outlined by the European Community Council Directive 2010/63/EU [22]. The brain cortex slices (200 mg) were cut and accurately weighed before homogenization. The studied group consisted of brain slices which were supplemented with the standard solution of NTs (each analyte at the concentration of 10 µg/g) (*n* = 6). The control samples were supplemented with an equal volume amount of MeOH (*n* = 6). For both groups, the procedures were equal: a tissue sample was homogenized on ice in 200 µL of cold 0.1% FA or 3% PCA solution in MeOH. The obtained homogenates were centrifuged (14,000 rpm, 10 min, 4 °C), and the supernatants were immediately separated from the precipitates. The homogenization was repeated two times, and the supernatants were combined. Then, 200 µL of the supernatant was processed with three different clean-up, preconcentration, and isolation methods: solid-phase extraction (SPE), solid-phase microextraction (SPME) or dispersive liquid–liquid microextraction (DLLME).

### 3.5. SPE, DLLME, and SPME Conditions

#### 3.5.1. SPE Settings

Because several options were chosen for the SPE procedure, the detailed conditions concerning the type of extraction column, conditioning procedure, and composition of the elution phase are presented in Table 2. The obtained residue was in each case dissolved in 50 μL of sample buffer (2 mM sodium tetraborate), centrifuged at 10,000 rpm for 5 min, and finally injected into the CE system. 

#### 3.5.2. DLLME Settings

An amount of 200 µL of the supernatant was mixed with 200 μL of ice-cold acetone using a vortex. The sample was shaken (5 min), then centrifuged (12,000 rpm, 5 min, 4 °C). Subsequently, 200 μL of the supernatant was mixed with 200 μL of ethanol and 200 μL of DCM using a vortex. The samples were shaken (10 min, speed: 850 rpm) and left for 10 min at room temperature in the dark, to separate the phases. The organic phase was taken and dried in a centrivap concentrator (45 °C, 30 min). Finally, the residue was dissolved in 50 µL of 2 mM sodium tetraborate, centrifuged at 10,000 rpm for 5 min, and next analyzed by the MEKC method.

#### 3.5.3. SPME Settings

The 96-well SPME procedure with a polystyrene–divinylbenzene (PS–DVB) resin and a 2.2 mL polypropylene deepwell storage plate was employed. The activation of SPME brushes was carried out with 1 mL of MeOH/H_2_O (1:1; *v/v*) for 30 min. Then, the brushes were washed with 1 mL of deionized water (10 s). The homogenates (200 µL) were diluted with water or a 0.1% FA water solution to 1 mL, loaded onto the SPME plate, and left for adsorption for 90 min at room temperature, while the tray was shaking (speed 850 rpm). Subsequently, the SPME brushes were rinsed with deionized water (10 s) to remove impurities. The desorption of the NTs was carried out with 1 mL of MeOH or a 1 mL solution of MeOH enriched with 20 ng/mL of IL (90 min, speed: 850 rpm, at room temperature). The desorption solution was further evaporated to dryness (centrivap, 45 °C, 1 h). Finally, the residue was dissolved in 50 µL of 2 mM sodium tetraborate, centrifuged at 10,000 rpm for 5 min, and analyzed by MEKC. 

### 3.6. Electrophoresis Equipment and MEKC Conditions

The CE system was equipped with an autosampler, a diode array detector (DAD), a temperature control device, and a data acquisition system supplied by the manufacturer (32 Karat 8.0). For our study, a previously optimized MEKC method for the separation and quantification of NTs was applied [9,19]. Uncoated fused silica capillary (75 µm i.d. 60.2 cm length) were employed, with UV detection (λ = 200 nm), hydrodynamic injection time of 8 s, applied voltage of 25 kV, temperature of 25.0 (±0.1) °C as tunable parameters. Between each run, the capillary was rinsed with 0.1 M NaOH for 1 min under the pressure of 50 psi and subsequently with Milli-Q water for 1 min under the pressure of 50 psi. The background electrolyte (BGE) consisted of 10 mM sodium tetraborate decahydrate, 30 mM SDS, 15% (*v/v*) MeOH, and 25 mM α-CD. The pH value was adjusted to pH 9.36 with 1 M NaOH.

## 4. Conclusions

The relatively simple procedures for the preparation of brain cortex slices, along with the short electromigration separation time, may offer a significant advantage over many other published methods, especially when long stages of sample preparation or derivatization procedures are involved. In this study, rapid clean-up procedures, based on three different extraction methods (SPE, DLLME, and SPME) in combination with the reliable MEKC–DAD technique for the simultaneous isolation of nine selected NTs from rat brain samples were tested and discussed. To the best of our knowledge, this is the first successful attempt to realize the off-line preconcentration SPME procedure with ionic liquid (1-ethyl-3-methyl imidazolium tetrafluoroborate) to improve the extraction efficiency of six NTs (DA, A, NA, l-Trp, l-Tyr, 5-HT) from brain samples. The proposed procedures can be successfully applied for the determination of this group of compounds, possessing great potential as a significant tool for clinical and neurological studies.

## Figures and Tables

**Table 1:** Signal intensities of the analytes obtained using different sample preparation protocols (each experiment was repeated six times *n* = 6).

	Homogenization Without Sample Clean-Up Procedure		Homogenization with FA/MeOH Followed by SPE SCX and Elution with			Homogenization with FA/MeOH Followed by SPE CSDAU and Elution with Analyte	Homogenization with FA/MeOH Followed by DLLME	Homogenization with FA/MeOH Followed by SPME with PS-DVB Coatings
	Homogenization Medium: FA/MeOH	Homogenization Medium: PCA/MeOH	FA/ACN/MeOH	MeOH	MeOH with NH_4_OH (pH 9.3)	MeOH		
	Peak Height (mean) ± SD							
**VMA**	3642.8 ± 78	1020.0 ± 101	55,729.2 ± 150	n.d.	n.d.	2045.3 ± 27	n.d.	n.d.
**HVA**	3789.4 ± 86	450.3 ± 26	59,667.0 ± 640	n.d.	n.d.	1568.7 ± 95	n.d.	n.d.
**DA**	n.d.	n.d.	n.d.	240.3 ± 19	54.3 ± 9	n.d.	8537.0 ± 84	15,043.3 ± 140
**A**	n.d.	n.d.	n.d.	1002.0 ± 80	105.7 ± 15	n.d.	2100 ± 25	2732 ± 28
**NA**	n.d.	n.d.	n.d.	102.7 ± 10	n.d.	n.d.	2543 ± 32	8964.2 ± 77
**l-Trp**	n.d.	n.d.	n.d.	1403.6 ± 42	n.d.	n.d.	n.d.	4667 ± 38
**l-Tyr**	n.d.	n.d.	n.d.	3578.5 ± 56	104.3 ± 19	n.d.	5462 ± 88	6098 ± 62
**5-HT**	1890.7 ± 120	n.d.	94.7 ± 12	1638.4 ± 68	59.0 ± 8	582.5 ± 35	9589 ± 94	5516 ± 49

**Notes:** FA/MeOH—0.1% formic acid in methanol; PCA/MeOH—3% perchloric acid in methanol; FA/ACN—0.1% formic acid in acetonitrile; PS-DVB—polystyrene–divinylbenzene; NH_4_OH—ammonium hydroxide; SPE SCX—solid-phase extraction strong cation exchange; SPE CSDAU—solid-phase extraction clean screen DAU; DLLME—dispersive liquid–liquid microextraction; SPME—solid-phase microextraction; VMA—vanillylmandelic acid; HVA—homovanillic acid; DA—dopamine; A—adrenaline; NA—noradrenaline; l-Trp—l-tryptophan; l-Tyr—l-tyrosine; 5-HT—serotonin, n.d.—not detected.

**Table 2:** SPE settings for the clean-up and preconcentration of NTs from brain sample homogenates (200 µL of sample). The SPE columns were washed each time with 1 mL of H_2_O, and the eluents were evaporated to dryness (45 °C/1 h) (each experiment was repeated six times *n* = 6).

Type of SPE Resin	Column Conditioning and Equilibration with a Mixture of	Elution With 2 × 200 μL of
Strong Cation Exchange (SCX)	1 mL 0.1% FA in ACN (apparent pH 3), 1 mL MeOH, 1 mL H_2_O	MeOH or 0.1% FA in ACN/MeOH (1:1, *v/v*) or MeOH + 0.2 M NH_4_OH
Clean Screen DAU (CSDAU)	1 mL MeOH, 1 mL H_2_O (pH 7)	MeOH + 0.2 M NH_4_OH

## Abbreviations

NTs	Neurotransmitters
CNS	Central nervous system
IL-SPME	Ionic liquid solid-phase microextraction
DA	Dopamine
A	Adrenaline
NA	Noradrenaline
5-HT	Serotonin
l-Trp	l-tryptophan
l-Tyr	l-tyrosine
IL	Ionic liquid
MEKC	Micellar electrokinetic chromatography
FA	Formic acid
PCA	Perchloric acid
MeOH	Methanol
DAD	Diode array detector
HVA	Homovanillic acid
VMA	Vanillylmandelic acid
LOD	Limit of detection
CE	Capillary electrophoresis
BGE	Background electrolyte
SPE SCX	Solid-phase extraction strong cation exchange
CS DAU	Clean Screen DAU
DLLME	Dispersive liquid–liquid microextraction
PS-DVB	Polystyrene–divinylbenzene
ACN	Acetonitrile
LIF	Laser-induced fluorescence
MS	Mass spectrometry
MTs	Migration times
l-DOPA	l-dihydroxyphenylalanine
SDS	Sodium dodecyl sulfate
DCM	Dichloromethane
α-CD	α-Cyclodextrin
